# Effects of challenge with *Clostridium perfringens*, *Eimeria* and both on ileal microbiota of yellow feather broilers

**DOI:** 10.3389/fmicb.2022.1063578

**Published:** 2022-12-01

**Authors:** Xin Feng, Tonghao Li, Hui Zhu, Lidan Liu, Shengqun Bi, Xiaolin Chen, Huihua Zhang

**Affiliations:** ^1^School of Life Sciences and Engineering, Foshan University, Foshan, China; ^2^Foshan Zhengdian Biology Technology Co., Ltd., Foshan, China

**Keywords:** challenge, *C. perfringens*, *Eimeria*, microbiota, yellow feather broiler

## Abstract

In the poultry industry worldwide, *Clostridium perfringens* has been causing major economic loss as it can cause necrotic enteritis (NE). The coccidial infection has been considered as the most important predisposing factor of NE caused by *C. perfringens*. In this study, we aimed to advance our knowledge on ileal microbiota of yellow feather broilers under *C. perfringens* and/or *Eimeria* challenge. Total of 80 healthy day old yellow feather broilers were randomly assigned to four groups including: Control, *C. perfringens* challenge group (C. Per), *Eimeria* challenge group (Cocc), and *C. perfringens* plus *Eimeria* challenge group (Comb). On day 14, the Cocc and Comb group broilers were orally gavaged 1 ml PBS solution containing 25,000 oocysts of *Eimeria brunetti* and 25,000 oocysts of *Eimeria maxima*. Starting on day 17, the C. Per and Comb group broilers were orally gavaged 10 mL of *C. perfringens* per bird (4 × 10^7^ CFU/mL, ATCC^®^ 13124™ Strain) every day for 6 days. 16S rRNA gene sequencing was performed on extracted DNA of ileal digesta samples. The results showed that *C. perfringens* alone did not affect the alpha diversity of ileal microbiome in yellow feather broilers but co-infection with *Eimeria* significantly decreased the diversity of ileal microbiota. *C. perfringens* and *Eimeria* challenge also decreased the relative abundance of beneficial bacteria including *Bacteroidetes* at the phylum level and *Faecalibacterium* at the genus level. At the species level, the relative abundance of *Candidatus Arthromitus* was significantly decreased in the *Eimeria* challenged groups. This microbial shift information of ileal microbiota under *C. Perfringens* and *Eimeria* challenge provide important reference data for the development of therapeutic approaches to necrotic enteritis in yellow-feather broiler chickens.

## Introduction

*Clostridium perfringens* (*C. perfringens*) is a constituent of normal flora in the digestive tract of animals and humans (Miller et al., 2010). In the poultry industry worldwide, *C. perfringens* has been causing major economic loss as it can cause necrotic enteritis (NE). Clinical or subclinical NE usually occurs in broiler chickens between the ages of 2–6 weeks (Skinner et al., 2010). In healthy chickens, *C. perfringens* almost always exist at levels less than 10^5^ CFU/g intestinal content (Caly et al., 2015). Predisposing factors including high levels of dietary non-starch polysaccharide grains or fish meal proteins, physiological stress, *Fusarium* mycotoxins in feed as well as coccidial infection normally exist to induce the outbreak of NE (Timbermont et al., 2011; Moore, 2016; Zaytsoff et al., 2020).

The coccidial infection has been considered the most important predisposing factor of NE caused by *C. perfringens*. The species of obligate intracellular parasites of the genus *Eimeria* can cause potentially severe enteritis (Attree et al., 2021), resulting significantly economic loss to the poultry industry. Four species of *Eimeria*, including *E. acervulina*, *E. maxima*, *E. necatrix*, and *E. tenella*, are considered most important due to their pathogenicity, global prevalence, and overall economic impact (Attree et al., 2021). When co-infected with *Eimeria*, the NE incidence and the mortality rate of chickens are higher (Baba et al., 1992). *Eimeria* can cause damage to the epithelium or induce mucogenesis, thus providing a niche for *C. perfringens* colonization and proliferation (Collier et al., 2008; Van Immerseel et al., 2009). Infectious dose, age, and immune status of the host could all affect the response of the chicken to coccidial infection, ranging from few clinical signs or reduction in weight gain, feed conversion, or egg production to severe enteritis and death (Attree et al., 2021). Coccidiosis prevention would be one way to reduce the incidence of NE caused by *C. perfringens.*

Due to the significant economic loss and compromised animal welfare caused by *C. perfringens* and *Eimeria*, effective control of NE necessitates explorations of alternative strategies due to chemoprophylaxis resistance and limited cost-effective vaccines (Giannenas et al., 2012; Ritzi et al., 2014). In 2020, the ban of antibiotic growth promoters in China has resulted in re-emergence of NE in poultry industry. Gut microbial shift plays a role in the progress of disease development. It was reported that NE development in chickens is associated with microbial shift within the GI tract (Kim et al., 2015). It has not been clear if microbial shift is a predisposing factor or more of a consequence of NE. To prevent *C. perfringens* and *Eimeria* infection and improve the gut health of broilers, the gut microbial information of infected chickens is important to know. In this study, we aimed to advance our knowledge on ileal microbiome of yellow feather broilers under *C. perfringens* and/or *Eimeria* challenge and provide reference data for future therapeutic strategies for disease control.

## Materials and methods

### Ethics statement

This experimental protocol was approved by the Ethical Committee and conducted under the supervision of the Institutional Animal Care and Use Committee of Foshan University (Foshan, China).

### Experimental design and sampling

Total of 80 healthy day old yellow feather broilers were randomly assigned to four groups including: Control, *C. perfringens* challenge group (C. Per), *Eimeria* challenge group (Cocc), and *C. perfringens* plus *Eimeria* challenge group (Comb). Birds under different treatments were raised in different pens. On day 14, the Cocc and Comb groups broilers were orally gavaged 1 mL mixed-species *Eimeria* oocysts solution. The mixed-species *Eimeria* spp. PBS-based solution contained 25,000 oocysts of *Eimeria brunetti* and 25,000 oocysts *E. maxima*. Starting on day 17, the C. Per and Comb groups broilers were orally gavaged 10 mL of *C. perfringens*/bird (4 × 10^7^ CFU/mL; *Clostridium perfringens* ATCC^®^ 13124™ Strain; cultured in Reinforced Clostridium Medium) everyday for 6 days. Fot the control groups birds, 1 ml of sterile PBS solution was orally gavaged. On day 24, six broilers randomly selected from each group were sacrificed by cervical dislocation and exsanguinated. The ileal digesta was collected from each broiler and immediately placed into a 2 mL Eppendorf tube. The digesta samples were stored at −80°C for later analysis.

The facility was thoroughly cleaned and disinfected before the bird placement. Temperature was maintained at 33–34°C initially and gradually decreased until 22–24°C by the third week. During the study, all the birds had free access to the same feed and clean water.

### Ileal digesta DNA extraction and microbiota data analysis

The DNA extraction and high-throughput sequencing analysis for this study were the same as our previous study (Feng et al., 2020). Briefly, total genome DNA from ileal digesta was extracted using the Cetyltrimethyl Ammonium Bromide method. Extracted DNA was monitored on 1% agarose gels before being diluted to 1 ng/μL to prepare amplicons for high-throughput sequencing. Conventional PCR was used to amplify the V3-V4 regions of the 16S rRNA genes using primers 515F (5′- GTGYCAGCMGCCGCGGTAA-3′) and 806R (5′-GGACTACNNGGGTATCTAAT-3′). The PCR reaction mix consisted of 15 μL of Phusion^®^ High-Fidelity PCR Master Mix (New England Biolabs, MA, USA), 0.2 μM of forward and reverse primers, and about 10 ng template DNA. Sequencing libraries were generated using TruSeq^®^ DNA PCR-Free sample preparation kit (Illumina, San Diego, CA, USA). The library quality was assessed on a Qubit @ 2.0 Fluorometer (Thermo Fisher Scientific, MA, USA) and Agilent Bioanalyzer 2100 system (Agilent Technologies, Inc., Santa Clara, CA, USA). The bar-coded amplicons were sequenced on an Illumina NovaSeq system and 250 bp paired-end reads were generated.

Paired-end reads were merged using Fast Length Adjustment of Short reads software (FLASH; V1.2.11) to obtain raw tags. Then quality control was conducted using fastp software to obtain high-quality clean tags. Finally, the Vsearch software was used to compare the clean tags with the database to detect and remove chimeras to obtain the effective tags (Haas et al., 2011). The obtained effective tags were denoised using the DADA2 module in QIIME2. Sequences with an abundance less than five were filtered out to obtain the final amplicon sequence variants (ASVs). Subsequently, the obtained ASVs were compared with the database (Silva138.1) using the classify-sklearn module in QIIME2 to obtain the species information of each ASV. Alpha diversity analysis (shannon, simpson, chao1, goods coverage, dominance, and pielou) and Beta diversity analysis were conducted in QIIME2. Principal component analysis (PCA) plot was generated using the “ade4” and “ggplot2” packages of the R software (v. 3.5.3).^1^ Differentially abundant genera among groups were identified using linear discriminant analysis (LDA) effect size (LEfSe) analysis (Segata et al., 2011). Phylogenetic Investigation of Communities by Reconstruction of Unobserved States (PICRUSt2) was performed to make inferences about the metabolic functions of the microbial community and metagenome metabolic functions were assessed using the KEGG orthology database (Douglas et al., 2020).

### Statistical analysis

Alpha diversity index and taxonomic data were analyzed using the PROC GLIMMIX procedure of SAS (SAS Institute, Inc., Cary, NC, USA) including treatment as fixed effect in the model. The significance was declared at *P* < 0.05 and trends at *P* < 0.1. Tukey multiple comparison method was used to detect difference between two groups when treatment effect was significant.

## Results

### Similarity analysis and alpha diversity

**FIGURE 1:**
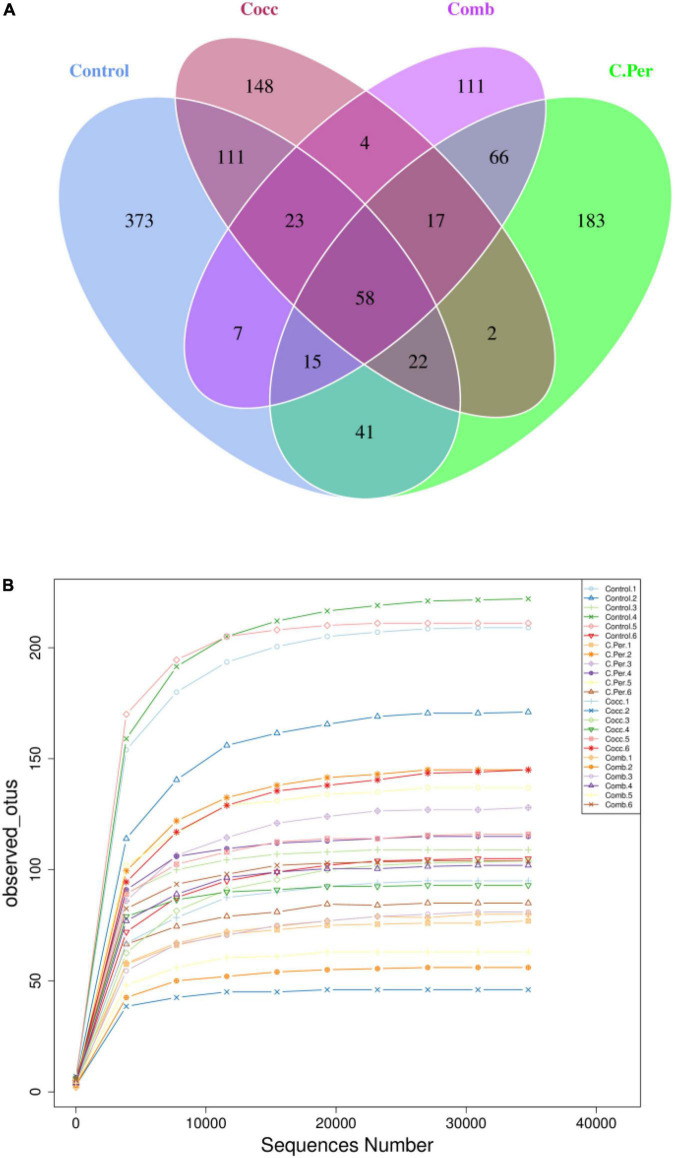
Number of Amplicon Sequence Variants (ASVs) in each group. **(A)** Venn diagram of shared and specific ASVs in the four groups. **(B)** Rarefaction curve of observed otus in all samples. C. Per, *C. perfringens* challenge; Cocc, *Eimeria* challenge; Comb, *C. perfringens* and *Eimeria* challenge.

**FIGURE 2:**
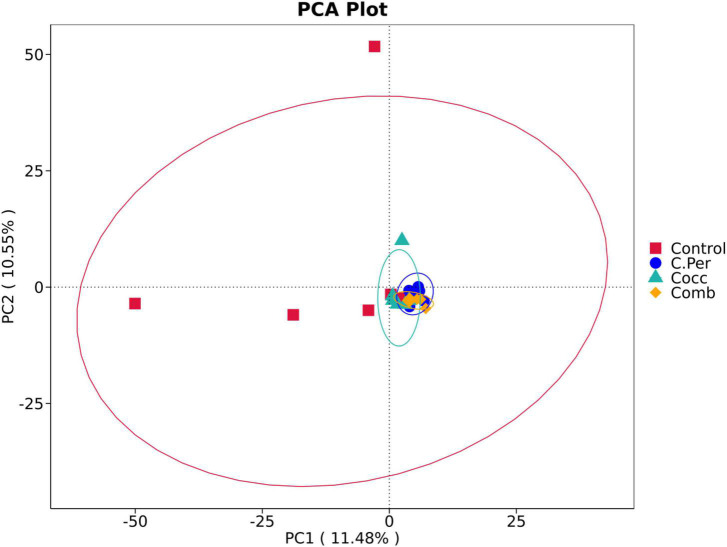
Principle component analysis of the ileal microbiota in different groups. C. Per, *C. perfringens* challenge; Cocc, *Eimeria* challenge; Comb, *C. perfringens* and *Eimeria* challenge.

To evaluate how infection of *C. perfringens* and *Eimeria* shift the microbial composition of chickens, 16S rRNA gene sequencing was performed on extracted DNA of ileal digesta samples. Total number of Amplicon Sequence Variants (ASVs) in all four groups was 1,185 and 58 ASVs were shared by all groups (Figure 1A). Compared to the control group, all the other three groups had lower number of group specific ASVs (control 373 vs. C. Per 183, Cocc 148, and Comb 111) indicating lower diversity of bacterial species. Rarefaction curve of observed otus revealed that there was sufficient sequence coverage to describe the bacterial composition of each group (Figure 1B). Principal component analysis revealed that the first and second principal components explained 11.48 and 10.55% of the variation among samples, respectively (Figure 2). Samples from different groups could not be clearly separated from each other. However, it can be seen from the PCA plot that the three groups under challenge clustered together. Effects of *C. Perfringens* and *Eimeria* challenge on alpha diversity of ileal microbiota in yellow feather broilers are present in Table 1. Compared to the control group, challenge with *C. perfringens* alone did not affect alpha indices including Shannon, and Chao1. But *Eimeria* challenge alone or combined infection with *C. perfringens* all significantly decreased the values of these indices (*P* < 0.05). The indices of Simpson, Pielou, and Dominance were not different among four groups (*P* > 0.05).

**TABLE 1:** Effects of *Clostridium perfringens* and/or *Eimeria* challenge on alpha diversity indices of ileal microbiota in yellow-feather broilers.

Items	Control	C. Per	Cocc	Comb	SEM	*P*
Shannon	3.56^a^	2.98^a^^b^	2.41^b^	2.39^b^	0.293	0.026
Simpson	0.79	0.72	0.64	0.65	0.059	0.28
Chao1	171.9^a^	115.1^a^^b^	100.2^b^	81.6^b^	14.52	0.0018
Pielou	0.48	0.44	0.37	0.38	0.039	0.16
Dominance	0.21	0.27	0.36	0.35	0.059	0.28

C. Per, *C. perfringens* challenge; Cocc, *Eimeria* challenge; Comb, *C. perfringens* and *Eimeria* challenge. Within a row, means without a common superscript (a, b) differ (*P* < 0.05).

### Taxonomic composition of ileal microbiota

**FIGURE 3:**
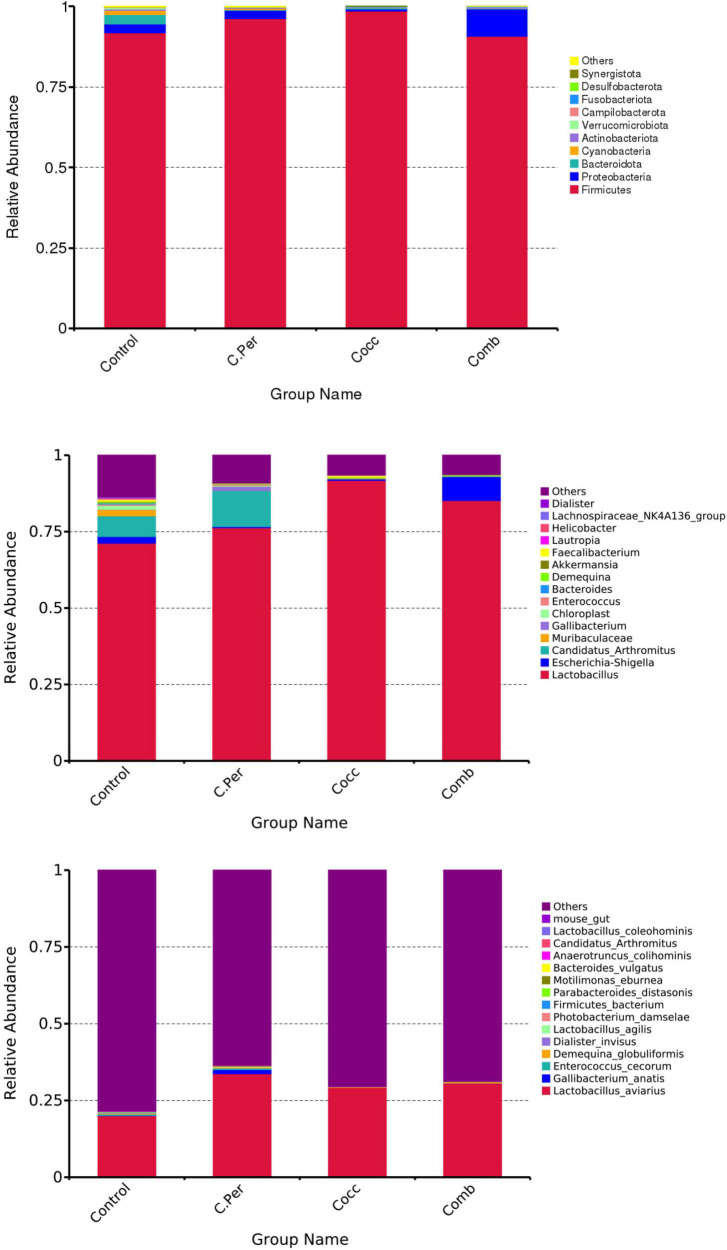
Phylum-level, genus-level, and species level taxonomic composition of the ileal bacterial communities in four groups. C. Per, *C. perfringens* challenge; Cocc, *Eimeria* challenge; Comb, *C. perfringens* and *Eimeria* challenge.

The main phyla present in the ileal digesta microbiota of the birds are shown in Table 2 and Figure 3. It was observed that *Firmicutes* and *Bacteroidetes* were the most frequent phyla, regardless of the treatment. Among the top 10 phyla, only the relative abundance of *Bacteroidetes* and *Desulfobacterota* were different among four groups. The *C. perfringens* and/or *Eimeria* challenge significantly decreased the relative abundance of *Bacteroidetes* compared to the control group (*P* < 0.05). In the ileal microbiota of yellow feather broilers, *Bacteroidetes* has the third highest abundance. At the genus level, among the top 15 genera analyzed, the relative abundance of *Faecalibacterium* was lower in the C. Per and Comb group in comparison with the control group. No difference between the control group and Cocc group was observed regarding the relative abundance of *Faecalibacterium* (Table 3). The relative abundance of the top 15 species in the ileal digesta microbiota of the broilers is shown in Table 4. The *Lactobacillus aviarius* was the most abundant species and its relative abundance was not different among the four groups (*P* = 0.90). Compared to the control group, the C. Per group had higher relative abundance of *Motilimonas eburnea* (*P* = 0.04). Its relative abundance in Cocc and Comb groups were not different from the control group. The control and C. Per groups had significantly higher abundance of *Candidatus Arthromitus* than the Cocc and Comb groups (*P* = 0.018).

**TABLE 2:** Effects of *Clostridium perfringens* and/or *Eimeria* challenge on taxonomic composition of ileal microbiota at phylum level in yellow-feather broilers.

Items,%	Control	C. Per	Cocc	Comb	SEM	*P*
*Firmicutes*	91.77	96.26	98.70	90.79	4.014	0.47
*Proteobacteria*	2.74	2.67	0.62	8.43	3.867	0.53
*Bacteroidetes*	3.07^a^	0.16^b^	0.37^b^	0.18^b^	0.760	0.03
*Cyanobacteria*	1.25	0.27	0.02	0.0008	0.478	0.24
*Actinobacteriota*	0.52	0.57	0.22	0.55	0.248	0.72
*Verrucomicrobiota*	0.24	0.02	0.05	0.01	0.117	0.49
*Campilobacterota*	0.21	0.015	0.007	9.58E-6	0.080	0.21
*Fusobacteriota*	0.06	0.013	0.013	0.012	0.033	0.65
*Desulfobacterota*	0.07^a^	0.001^a^^b^	0.002^a^^b^	0^b^	0.017	0.03
*Synergistota*	0.016	0	0.004	0	0.008	0.49
Others	0.06	0.02	1.11E-16	0.05	0.016	0.09

C. Per, *C. perfringens* challenge; Cocc, *Eimeria* challenge; Comb, *C. perfringens* and *Eimeria* challenge. Within a row, means without a common superscript (a, b) differ (*P* < 0.05).

**TABLE 3:** Effects of *Clostridium perfringens* and/or *Eimeria* challenge on taxonomic composition of ileal microbiota at genus level in yellow-feather broilers.

Items,%	Control	C. Per	Cocc	Comb	SEM	*P*
*Lactobacillus*	71.25	76.33	91.79	85.19	6.498	0.15
*Candidatus_Arthromitus*	6.77	11.79	0.08	0.17	4.242	0.18
*Muribaculaceae*	2.03	0.02	0.19	0	0.679	0.13
*Escherichia_Shigella*	2.18	0.36	0.48	7.83	3.762	0.47
*Gallibacterium*	0.11	1.36	1.95*E* − 18	0.01	0.573	0.30
*Chloroplast*	1.24	0.27	0.018	0.008	0.478	0.25
*Enterococcus*	0.45	0.48	0.008	0.06	0.198	0.23
*Bacteroides*	0.37	0.002	0.06	0.01	0.129	0.19
*Demequina*	0.29	0.27	0.18	0.30	0.152	0.94
*Akkermansia*	0.22	4.8*E* − 6	0.04	0	0.109	0.45
*Faecalibacterium*	0.76^a^	0.04^b^	0.59^a^	0.07^b^	0.077	< 0.0001
*Lautropia*	0.19	0.007	0.06	0.003	0.097	0.51
*Helicobacter*	0.21	0.006	0.003	0	0.081	0.22
*Lachnospiraceae_NK4A136_group*	0.21	0	0.004	0	0.080	0.19
*Dialister*	0.16	0.03	0.04	0.02	0.085	0.59
Others	13.55	9.05	6.44	6.31	3.093	0.33

C. Per, *C. perfringens* challenge; Cocc, *Eimeria* challenge; Comb, *C. perfringens* and *Eimeria* challenge. Within a row, means without a common superscript (a, b) differ (*P* < 0.05).

**TABLE 4:** Effects of *Clostridium perfringens* and/or *Eimeria* challenge on taxonomic composition of ileal microbiota at species level in yellow-feather broilers.

Items,%	Control	C. Per	Cocc	Comb	SEM	*P*
*Lactobacillus_aviarius*	20.05	33.74	29.30	30.66	13.65	0.90
*Gallibacterium_anatis*	0.11	1.36	2.17*E* − 17	0.01	0.572	0.29
*Enterococcus_cecorum*	0.45	0.46	0.007	0.056	0.199	0.23
*Demequina_globuliformis*	0.27	0.24	0.16	0.27	0.137	0.94
*Dialister_invisus*	0.16	9.58*E* − 4	0.04	4.34*E* − 17	0.084	0.48
*Photobacterium_damselae*	0.004	0.20	0	0.14	0.074	0.18
*Lactobacillus_agilis*	0.08	0.23	1.08*E* − 17	0.01	0.069	0.09
*Firmicutes_bacterium*	0.11	0	0.006	2.17*E* − 17	0.057	0.43
*Parabacteroides_distasonis*	0.10	0	0.01	0	0.051	0.44
*Motilimonas_eburnea*	0.006^a^	0.15^b^	0.005^a^	0.084^a^^b^	0.040	0.04
*Bacteroides_vulgatus*	0.07	0	0.01	0	0.033	0.38
*Anaerotruncus_colihominis*	0.07	0.002	0.03	0	0.023	0.17
*Candidatus_Arthromitus*	6.76^a^	11.79^a^	0.078^b^	0.17^b^	4.242	0.018
*Lactobacillus_coleohominis*	0.06	0.03	0.02	0.03	0.021	0.43
*mouse_gut*	0.03	0	0.007	0	0.013	0.49
Others	78.43	63.51	70.40	68.74	13.79	0.89

C. Per, *C. perfringens* challenge; Cocc, *Eimeria* challenge; Comb, *C. perfringens* and *Eimeria* challenge. Within a row, means without a common superscript (a, b) differ (*P* < 0.05).

**FIGURE 4:**
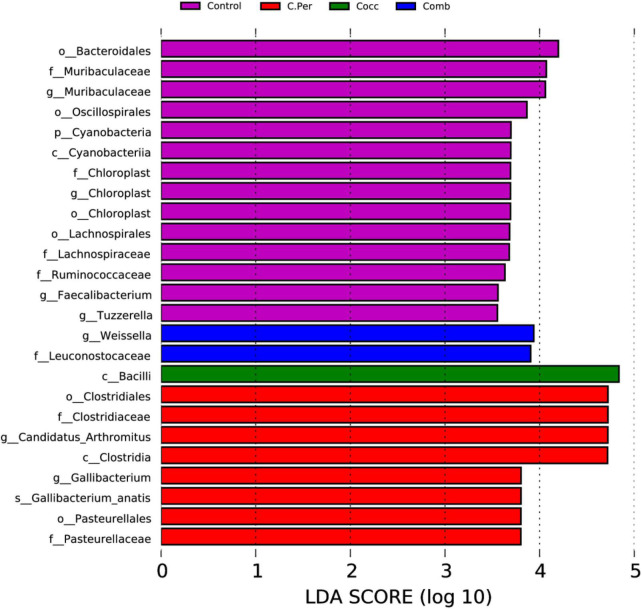
linear discriminant analysis (LDA) effect size (LEfSe) Analysis of differential species among all groups shown in the LDA value distribution histogram. The presented species are biomarkers with statistical differences between groups (LDA > 3.5). The length of the histogram (LDA score) represents the impact of the different species.

Linear discriminant analysis (LDA) effect size analysis (LDA > 3.5) was performed to discriminate the differences on the community composition between groups. A total of 25 biomarkers bacterial genera were identified in the four groups (Figure 4). *Bacteroidales*, *Muribaculaceae* (both at the family and genus level), *Oscillospirales*, *Cyanobacteria*, *Cyanobacteria*, *Chloroplast* (at the order, family, and genus levels), *Lachnospirales*, *Lachnospiraceae*, *Ruminococcaceae* were enriched in the control group. The C. Per group was enriched with *Clostridiales*, *Clostridiaceae*, *Candidatus Arthromitus*, *Clostridia*, *Gallibacterium*, *Gallibacterium anatis*, *Pasteurellales*, and *Pasteurellaceae*. In addition, *Bacilli* was enriched in Cocc group and *Weissella* and *Leuconostocaceae* were enriched in the Comb group. Consistent with results from Table 2, the control group had the highest abundance of *Cyanobacteria*, but not statistically different compared with other groups. This also applies to *Candidatus Arthromitus*, *Muribaculaceae*, *Gallibacterium*, and *Chloroplast* meaning that the groups in which they were enriched had the highest abundance but not significantly different from other groups. Based on the LEfSe analysis, *Faecalibacterium* was enriched in the control group. The control group did have significantly higher abundance of *Faecalibacterium* compared to the C. Per and Comb group (*P* < 0.05), but not different from the Cocc group.

### Function prediction using phylogenetic investigation of communities by reconstruction of unobserved states

**FIGURE 5:**
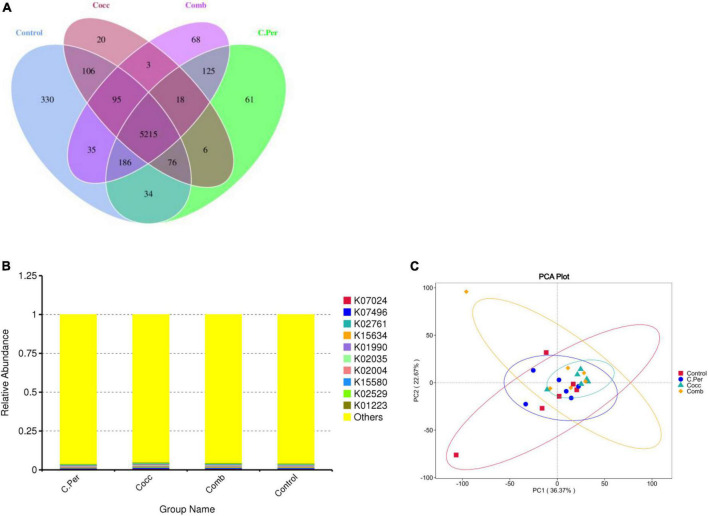
Function predictions using phylogenetic investigation of communities by reconstruction of unobserved states (PICRUSt2). **(A)** Venn diagram of shared and specific functions predicted in the four groups. **(B)** Barplot of the top functions predicted against KEGG orthology (KO) database. **(C)** Principle component analysis (PCA) of the functions predicted in different groups; C. Per, *C. perfringens* challenge; Cocc, *Eimeria* challenge; Comb, *C. perfringens and Eimeria challenge.*

The predicted functions against the KEGG orthology database are presented in Figure 5. Total of 6,378 KOs were predicted among four groups and 5,215 were shared by all groups (Figure 5A). Compared to the control group, the other three groups had lower number of group specific microbial functions (330 for control vs. 61 for C. Per, 20 for Cocc, and 68 for Comb). The relative abundance of the top 10 microbial functions (Figure 5B) are most involved in the metabolism (K07024, K02761, K15634, and K01223), Cellular Processes (K02035 and K15508); genetic information processing (K02529 and K07496), and Environmental Information Processing (K01990 and K02004). All these functions were not statistically different among the four groups. The principal component analysis plot showed clusters of samples based on their microbial function similarity. It can be seen that samples from four groups could not be separated completely. The first and second components explained 36.37 and 22.67% of the variation, respectively (Figure 5C).

## Discussion

Necrotic enteritis (NE) is a disease of the small intestine of chickens caused by *C. perfringens* (Zaytsoff et al., 2020). When *C. Perfringens* colonizes and proliferates in the small intestine, extracellular toxins produced can damage the intestinal wall thus cause necrotic enteritis. Modulation of *C. perfringens* on intestinal tight junctions and host immune response was reported earlier (Mitchell and Koval, 2010; Daneshmand et al., 2022). Predisposing factors such as high dietary protein or *Eimeria* infection have been utilized in the experimental reproduction of NE to investigate its pathogenesis or prevention (Shojadoost et al., 2012). Physiological stress as predisposing factor to NE has also been shown to increase densities of *C*. *perfringens* in the small intestine and weight gain impairment in chickens (Zaytsoff et al., 2020).

We used coccidia to facilitate an optimal NE reproduction in yellow feather broilers. During coccidiosis, *Eimeria* colonize the intestine and destroy intestinal epithelium cells which create an optimal environment for *C. perfringens* proliferation (Williams, 2005). In our study, lesions were not observed in the small intestine of the broilers inoculated with *C. perfringens* and/or *Eimeria*. Similarly, Wu et al. (2014) reported no observed lesions in the jejunum and ileum of *Eimeria* challenged birds. Daneshmand et al. (2022) observed that birds challenged with both *Eimeria* and *C. Perfringens* either on control diet or wheat-based diet had reduced performance coupled with enteric gross lesions and epithelial damage. The inconsistent findings might be related to the dose amount and *Eimeria* species used.

It has been shown that gut microbial community can alter the susceptibility of poultry to necrotic enteritis as they can affect gene expression in the ileum of the host (Tang et al., 2020). Six indices were often used to measure alpha diversity including Shannon, Simpson, Chao1, Good’s coverage, dominance, and Pielou. The greater the Chao1 and dominance indices, the higher the expected species richness of the microbiome. The greater the Shannon or the smaller the Simpson indices, the higher the diversity of the microbiome (Forbes et al., 2018). Pielou’s evenness index measures diversity along with species richness. A calculated value of Pielou’s evenness ranges from 0 (no evenness) to 1 (complete evenness). Compared to the control group, *C. perfringens* alone did not affect the richness or diversity of ileal microbiota. *Eimeria* challenge with or without *C. perfringens* all significantly decreased the Shannon and Chao1 indices indicating lower richness and diversity of intestinal microbiota in yellow feather broilers. Inconsistent to our results, increased chao1 index was observed in challenged birds (Bortoluzzi et al., 2019). *Eimeria* infection can significantly reduce the gut microbial diversity, most through reducing low abundance of operational taxonomic units (OTUs) and increasing dominance in the community (Perez et al., 2011). This was also observed in our study as groups challenged with *Eimeria* all had lower microbial diversity compared to the control group. Yang et al. (2021) investigated the link between ileal microbiota and disease severity in a chicken model of clinical NE. The authors reported that NE can significantly reduce the richness and Shannon index of ileal microbiota. Yang et al. (2019) reported that co-infection of *C. perfringens* and *Eimeria* significantly reduced species diversity in jejunal microbiota of broiler chicks but not cecal microbiota. Gastrointestinal tract is highly complex with numerous bacterial species, which would all contribute the development of NE. As shown in our results, changes in gut microbiota diversity induced by *Eimeria* may play an important role in predisposing the *C. Perfringens* infected birds to NE.

Healthy intestinal microbiota can enhance the host immune system and protection against intestinal pathogens (Ritzi et al., 2014). The dominance of beneficial microorganisms is essential to maintain gut homeostasis. Reduction of beneficial probiotic bacteria can predispose chickens to the onset of NE (Wu et al., 2014). LEfSe (Linear discriminant analysis Effect Size) determines the features most likely to explain differences between groups (Segata et al., 2011). Based on the LEfSe analysis, *Faecalibacterium* was enriched in the control group. Statistical analysis showed that the control group had significantly higher relative abundance of *Faecalibacterium* compared to the C. Per and Comb group. Surprisingly, no difference between the control group and Cocc group was observed. The sole known species of *Faecalibacterium*, *Faecalibacterium prausnitzii*, is a probiotic and its decline is associated with the development of chronic inflammation (Maioli et al., 2021). The decreased relative abundance of *Faecalibacterium* in the C. Per and Comb group indicated possible intestinal inflammation after challenge with *C. perfringens*. In chickens with severe NE, *Firmicutes* was significantly decreased whereas *Proteobacteria* was increased (Yang et al., 2021). The increase in the relative abundance of pathogenic group indicates that the birds are carriers of pathogenic bacteria, which might lead to future health issues (Akerele et al., 2022). As stated previously, in our study no lesions are observed in our study and the relative abundance of *Firmicutes* and *Proteobacteria* were not affected either. Bortoluzzi et al. (2019) observed no difference on the relative abundance of the most abundant phyla (*Firmicutes*, *Bacteroidetes*, and *Proteobacteria*) between the challenged and unchallenged birds. But in our study, the challenge groups had lower abundance of *Bacteroidetes* which was inconsistent to Bortoluzzi et al. (2019). The *Bacteroidetes* phylum has the ability to degrade a wide range of complex carbohydrates, making its dominance in many diverse environments (McKee et al., 2021). Decreased abundance of *Bacteroidetes* indicates lower ability of the ileal microbes on degradation of complex carbohydrates. Coccidial infection can dramatically decrease the resident microbiota and increase conditionally pathogenic bacteria such as *Clostridiales* (Ramanan et al., 2016; Lu et al., 2021). It can lead to more severe clinical manifestations by providing an environment which is conducive for pathogenic bacteria, such as *Campylobacter jejuni*, *C. perfringens*, *Salmonella*, and other bacteria (Kogut et al., 1994; Ficko-Blean et al., 2012; Macdonald et al., 2019). Major perturbations in lactic acid-producing and butyrate-producing families of bacteria in chickens with NE were observed (Antonissen et al., 2016). However, the results have been inconsistent among studies due to the varying severity degree of NE. *Lactobacillus* is known as beneficial bacteria by competition with pathogens and produce lactic acid which can inhibit pathogenic bacteria (Belenguer et al., 2007). *Lactobacillus* was more abundant in the upper GI tract compared with the lower tract. *C. Perfringens* challenge could decrease the *Lactobacillus* population in ileum (Antonissen et al., 2016; Akerele et al., 2022). But this was not observed in our study. *Lactobacillus reuteri*, *L. johnsonii*, *L. acidophilus*, *L. crispatus*, *L. salivarius*, and *L. aviarius* were the predominant *Lactobacillus* species and present throughout the GI tract of chickens (Wang et al., 2014). Our results showed that at the species level, *L. aviarius* had the highest relative abundance which is similar to Gong et al. (2007)’s findings. *Lactobacillus aviarius* was first isolated from the intestine of chickens in Fujisawa et al. (1984) and is very useful enhancing immunity systems of the organisms (Al-Shaer et al., 2019). It has been reported that probiotics supplementation shifted bacterial community in broiler digestive tract, with *Lactobacillus salivarius* and *Lactobacillus aviarius* as dominant species in the treatment group. Some researchers reported suppressed *Lactobacillus* at the early stage of *C. perfringens* challenge (Fasina et al., 2016) whereas opposite findings were also reported by others (Liu et al., 2010). *C. perfringens* infection could suppress *L. aviarius* in the ileum of broilers (Du et al., 2015). In our study, the relative abundance of *Lactobacillus aviaries* was not different among all four groups. However, it should be noted that the variation of *Lactobacillus aviaries* abundance within groups was fairly high. *Lactobacillaceae* shift in broilers with NE induced by dual *E. maxima* and *C. perfringens* are dependent on breed whereas relative abundance of *Lactobacillus* was increasing in the ileal content of Cobb 500 broilers but decreasing in Ross 308 (Kim et al., 2015). In addition, the time of challenge may also play a role. In Yang et al. (2021)’s study, the chickens were inoculated with *E. maxima* on day 10 and 4 × 10^8^ CFU of *C. perfringens* on day 14. In our Comb group, we did this on day 14 and day 17. Rearing conditions could also affect the gut microbial compositions for NE challenged birds. Bortoluzzi et al. (2019) found that the cecal microbiota composition and function was more affected than the ileal microbiota for NE challenged bird raised in floor pens with reused litter compared to birds raised in cages. *Motilimonas eburnea* in the order *Alteromonadales*, was first isolated from coastal sediment and this novel species was named by the authors (Ling et al., 2017). The reason that the C. Per and Comb groups had higher abundance is not clear as limited research was reported on this species. *Candidatus Arthromitus*, also known as *Candidatus Savagella*, may provide a protective role in preventing the onset of the enteric condition in Turkeys (Hedblom et al., 2018). It has the potential to serve as an immune-stimulatory probiotic making it an organism of great interest to poultry researchers. Stanley et al. (2012) reported decreased abundance of *Candidatus Arthromitus* in the cecal digesta of broiler chickens challenged with oocysts of *E. acervulina*, *E. maxima*, and *E. brunette*. This was similar to our study as that the Cocc and Comb groups with *Eimeria* challenge had significantly lower abundance than the control and the C. Per groups.

Studying the composition of the gut microbiota not only can reflect the relationship between microorganisms and the host but also provide information of the microbial functions on the gut (Bortoluzzi et al., 2019). To further understand the functions performed by the gut microbes, we used PICRUSt2 to make inferences about the metabolic functions of the gut microbes against the KEGG orthology database. We found that the top 10 microbial functions are most involved in the metabolism (starch and sucrose metabolism), Cellular Processes (Quorum sensing), genetic information processing (transcription factors), and Environmental Information Processing (ABC transporters). All these predicted functions were not affected by treatments. Bortoluzzi et al. (2019) reported that NE mainly affected microbial functions of cecal microorganisms not ileal microorganism. They observed NE challenge enriched pathways related to DNA replication, proteins, amino acids related enzymes and metabolism, and transcription factors (Bortoluzzi et al., 2019). In that study, the challenged birds groups had enriched “ion channel” function compared to the control groups. We did not observe any difference regarding specific functions among the four groups and this might be because we analyzed the ileal digesta samples. Enriched pathways related to amino acid metabolism was observed in the challenged birds (Zhang et al., 2018; Yang et al., 2021). The possible reasons could be due to pathogens such as *Helicobacter pylori* and *Salmonella Typhimurium* which use amino acids as energy source (Zhang et al., 2018) or activated amino acid synthetic pathways in the remaining bacterial population to compensate for an inability of *C. perfringens* to synthesize amino acids (Yang et al., 2021). Yang et al. (2021) also suggested that metabolism of cofactors and vitamins may be enhanced in severe NE cases. In our study, the microbial functions were all predicted based on 16S rRNA sequencing data and follow-up metagenomics of the microbiota warrants further investigation.

Yellow-feather broiler, also known as three-yellow chicken (yellow feather, yellow skin, and yellow shank.) are high-quality chicken with excellent meat quality and flavor. They are mainly raised in south China. Researches on the inter relationship of NE and gut microbiota of yellow feather broilers were limited. An better understanding of the importance of gut microbiota in disease onset, progression, and treatment is utmost. The disease model would lay foundation for microbial manipulation of NE in yellow feather broilers. Further studies are needed to determine and improve the robustness and reproducibility of NE disease model.

## Conclusion

The present study used *Eimeria* for NE reproduction to evaluate the effects of *C. perfringens* on ileal microbiota of yellow feather broilers. In summary, *C. perfringens* alone did not affect the alpha diversity of ileal microbiota in yellow feather broilers but co-infection with *Eimeria* significantly decreased the Shannon and Chao1 indices. *Eimeria* or *C. perfringens* challenge also decreased the relative abundance of beneficial bacteria including *Bacteroidetes* at the phylum level, *Faecalibacterium* at the genus level and *Candidatus Arthromitus* at the species level.

## Data availability statement

The datasets presented in this study can be found in online repositories. The names of the repository/repositories and accession number(s) can be found below: Bioproject accession number: PRJNA892261.

## Ethics statement

The animal study was reviewed and approved by the Institutional Animal Care and Use Committee of Foshan University.

## Author contributions

XF, TL, and HZ conducted the experiment and wrote the manuscript together. LL and SB analyzed the samples. XC analyzed the data. HHZ provided critical feedback and helped shape the research. All authors contributed to the article and approved the submitted version.

## Footnotes

1
https://www.r-project.org/
